# Effect of Incisal Porcelain Veneering Thickness on the Fracture Resistance of CAD/CAM Zirconia All-Ceramic Anterior Crowns

**DOI:** 10.1155/2019/6548519

**Published:** 2019-08-26

**Authors:** Noha Badran, Sanaa Abdel Kader, Fayza Alabbassy

**Affiliations:** ^1^Fixed Prosthodontic Department, Faculty of Dentistry, Alexandria University, Alexandria City, Egypt; ^2^Dental Biomaterials Department, Faculty of Dentistry, Alexandria University, Alexandria City, Egypt

## Abstract

**Statement of Problem:**

In some clinical situations, the vertical length of either a prepared tooth or an implant abutment is short, while the occlusal clearance to be restored by a porcelain crown is large. Incisal thickness of the veneering porcelain should be considered to prevent mechanical failure of the crown.

**Purpose:**

The aim of this study is to evaluate the effect of two different incisal veneering porcelain thickness on the fracture resistance of the anterior all-ceramic CAD/CAM zirconia crown system as compared with the conventionally used metal ceramic crown system.

**Method:**

CAD/CAM zirconia all-ceramic and metal ceramic crowns were fabricated on the prepared dies with standardized dimensions and designs using standardized methods according to the manufacturer's instructions. All crowns were then adhesively luted with resin-based cement (Multilink cement system), subjected to thermal cycling and cyclic loading, and were loaded until fracture using the universal testing machine to indicate the fracture resistance for each crown material in each veneering thickness.

**Results:**

Statistical analysis was carried out, and the results showed that the fracture resistance of the nickel-chromium metal ceramic group was significantly higher than that of the CAD/CAM zirconia all-ceramic group. Also, the fracture resistance of crowns with 1.5 mm incisal veneering thickness was significantly higher than those with 3 mm incisal veneering thickness in both groups. Furthermore, there was no significant difference in the fracture mode of the two groups where 50% of the total specimens demonstrated Mode II (veneer chipping), while 35% demonstrated Mode I (visible crack) and only 15% demonstrated Mode III (bulk fracture).

**Conclusion:**

High failure load values were demonstrated by the specimens in this study, which suggest sufficient strength of both incisal veneering thickness in both crown systems to withstand clinical applications; however, the fracture patterns still underline the requirement of a core design that support a consistent thickness of the veneering ceramic, and it is recommended to conduct long-term prospective clinical studies to confirm findings reported in the present study.

## 1. Introduction

Since the late 1950s, the metal ceramic crown system has remained a standard modality for rehabilitation of anterior dentition, thanks to their good mechanical properties and to somewhat satisfactory esthetic results, along with a clinically acceptable quality of their marginal and internal adaptation [1, 2]. Longevity of metal ceramic complete coverage crowns, both in vivo and in vitro, has also been reported; however, veneering porcelain fracture remained a primary problem occurring in 5% to 10% of single-unit prostheses and represented the second most common cause of their replacement [3–8].

In the last 30 years, the growing patients' demand for highly esthetic and naturally appearing restorations has led to the development of new all-ceramic materials, whose mechanical characteristics have been dramatically improved to provide suitable longevity and limitation of the technical problems. Furthermore, a great advancement in dental ceramics has been achieved since the introduction of high-strength tetragonal yttria-partially stabilized zirconia (Y-TZP) which became the most interesting polycrystalline ceramic available for dentistry mainly due to the transformation-toughening mechanism [9, 10]. However, the opacity of zirconia limits its use to the fabrication of substructures, which are further veneered with high-translucent dental porcelain.

Porcelain compatibility is a concern on veneered zirconia restorations since recent studies on all-ceramic restorations reported a significant amount of porcelain chipping (15–62%), cracking (25–50%), delamination (less than 10.7%), and large fractures (3–33%) [11–14]. Several potential explanations for such fracture behavior have been reported, and one of these explanations was the veneering porcelain thickness.

So, it is obvious that restoration of the anterior teeth with crowns that have a framework for porcelain support is further complicated by the requirement, generally placed on the veneering porcelain, to simulate a lifelike tooth appearance. This is particularly challenging for anterior restorations where a high level of translucency, especially in the incisal and middle third of the tooth, is required, while the presence of the framework in these areas is thought to be necessary to provide mechanical resistance to fracture.

Therefore, this study was conducted to evaluate the effect of two different incisal veneering porcelain thicknesses on the fracture resistance of the anterior all-ceramic CAD/CAM zirconia crown system when comparing it with the conventionally used metal ceramic crown system.

## 2. Materials and Methods

### 2.1. Materials

The materials used in this study were as follows.

#### 2.1.1. Crown Materials

The description of the crown materials is shown in Table 1. The following are the two types of crown materials:

- All-ceramic crown materials
- Metal ceramic crown materials

#### 2.1.2. Luting Resin Cement

Universal self-curing resin-based system with the light-curing option (Multilink N system pack; Ivoclar Vivadent ACT, Benderstr, Liechtenstein) was used for cementation of both groups in this study.

#### 2.1.3. Aiding Materials and Devices

- Metal die (made of art alloy BF)
- Doublident (Doublident® W+D dental, P.O.B 508, D-25305 Elmshorn. Reference: WD 5080C; duplicating addition curing silicon)
- Epoxy resin material (RenCast® Epoxy casting Resin, Klybeckstrasse 200, CH. 4057 BASEL, Switzerland)
- CAD/CAM Zirkonzahn ceramic system
- Thermal cycling machine (made by Biomaterial Department, Faculty of Dentistry, Alexandria University)
- Custom made by the load cyclic machine (made by Biomaterial Department, Faculty of Dentistry, Alexandria University)
- Universal testing machine (Comten Industries Inc, St. Petersburg Florida, USA. Model no 942 D 10-20)

### 2.2. Methods

#### 2.2.1. Preparation of Master Die

**Figure 1:**
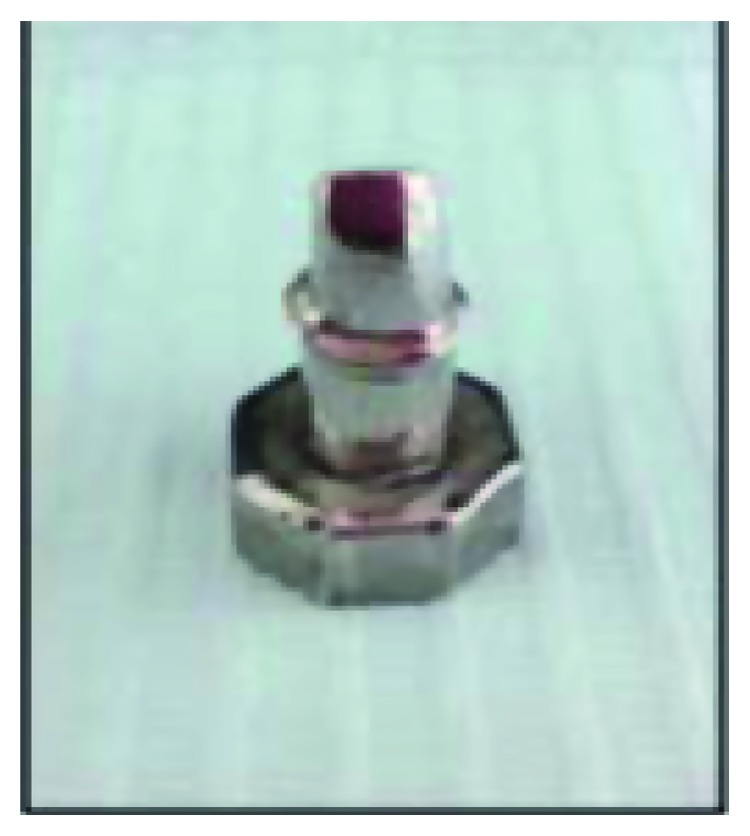
Stainless steel master die.

A specially designed stainless steel metal master die was milled to simulate the maxillary central incisor crown prepared to receive metal ceramic and all-ceramic full coverage crowns (Figure 1). The apicocoronal height of the prepared metallic die was 6 mm, with a mesiodistal dimension at the cervix of 5 mm, while the labiolingual dimension at the cervix was 4 mm and the width of the incisal edge was approximately 4 mm mesiodistally and 1.5 mm labiolingually. The axial wall taper was approximately 6 degrees on each side and 12 degrees total convergence angle. All transitions from the axial to the incisal edge were rounded and smooth.

#### 2.2.2. Duplication of the Master Die by Epoxy Resin

**Figure 2:**
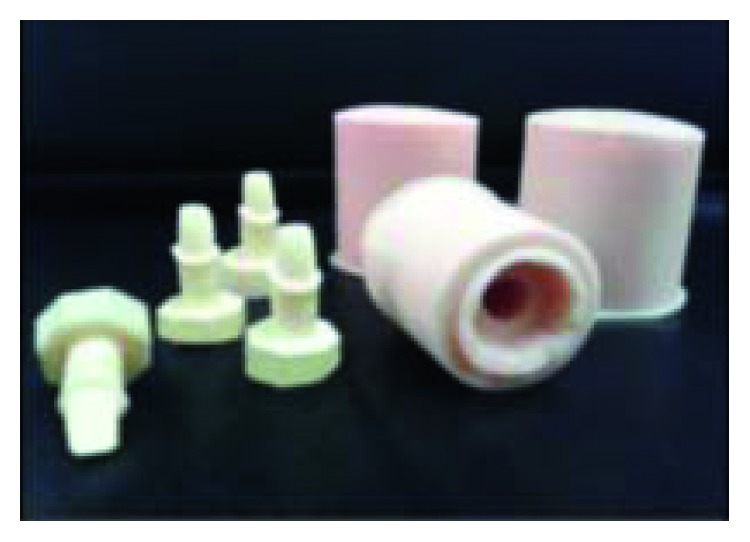
Epoxy resin replicas of master die.

Twenty negative replicas of the prepared master die were made by the duplicating addition silicon material and filled with the epoxy resin material having the same elastic modulus of dentin to get twenty positive replicas of the prepared maxillary central incisor and then the reproduced dies were smoothly polished (Figure 2).

#### 2.2.3. Grouping of the Specimens

The specimens were divided into two main groups (Table 2), each of ten according to the crown material.

 
*Group I*. CAD/CAM zirconia all-ceramic crowns (coping thickness of 0.5 mm).
 
*Group II*. Nickel-chromium metal ceramic crowns (coping thickness of 0.5 mm).
  Each group was subdivided into two subgroups according to the incisal veneering porcelain thickness.
 
*Subgroups Ia and IIa.* The incisal veneering porcelain thickness was 1.5 mm. *Subgroups Ib and IIb.* The incisal veneering porcelain thickness was 3 mm.

#### 2.2.4. Fabrication of Group I (All-Ceramic) Crowns

Copings of group I crowns were milled out from Zirkonzahn zirconia blanks in the following steps: optical impression of the specimens, designing of the coping using modeling software “Zirkonzahn modeler,” milling of zirconia blanks, and firing of the milled zirconia copings.

**Figure 3:**
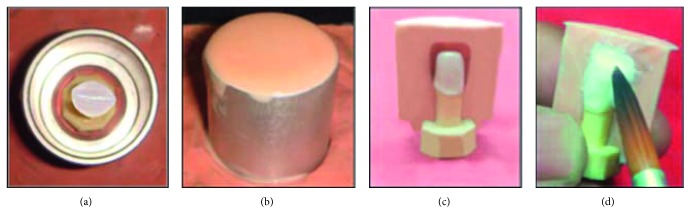
Making impression of full contoured CAD/CAM zirconia crown: (a) CAD/CAM zirconia coping inside the metal ring; (b) metal ring filled with the duplicating material; (c) split mold adapted on lined zirconia coping; (d) application of body porcelain and condensation.

**Figure 4:**
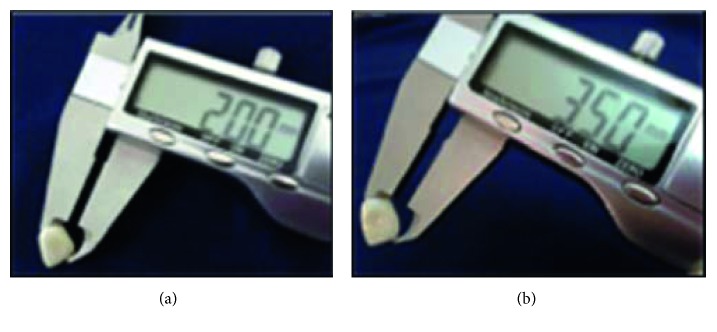
Checking thickness of the finished CAD/CAM zirconia all-ceramic crown: (a) all-ceramic crown of 2 mm incisal thickness; (b) all-ceramic crown of 3.5 mm incisal thickness.

Ten CAD/CAM zirconia copings were veneered with Ceramco®PFZ using the layering technique. To standardize the specimen's size, two extra specimens were milled to the full anatomical contour of maxillary central incisors, and the only difference was in the incisal edge thickness. One of the specimen had 2 mm incisal edge thickness (0.5 mm core, 1.5 mm veneer), and the other had 3.5 mm incisal edge thickness (0.5 mm core, 3 mm veneer); the thickness of both was checked using the Iwanson gauge. Two impressions were made for the full contoured crowns from duplicating addition silicon with the aid of a metal ring to reproduce two silicon molds (mold #1 and mold #2) which were then split along the long axis to be used as guidance during building of the porcelain veneering, and then all the specimens were fired according to the manufacturer's instructions (Figures 3(a)–3(d)). Each crown was checked for proper fit, thickness, and form, and finally all crowns were finished and glazed according to the manufacturer's instructions (Figures 4(a) and 4(b)).

#### 2.2.5. Fabrication of Group II (Metal Ceramic Crowns)

**Figure 5:**
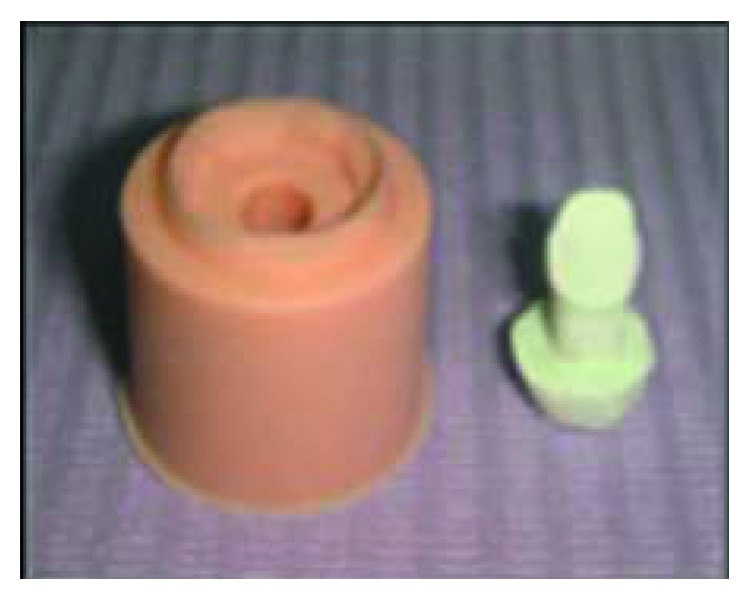
Poured stone die.

**Figure 6:**
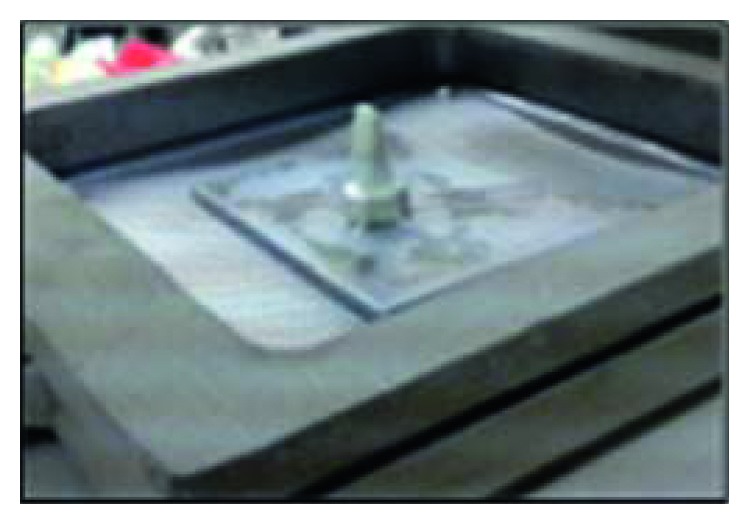
Vacuum formed resin template.

**Figure 7:**
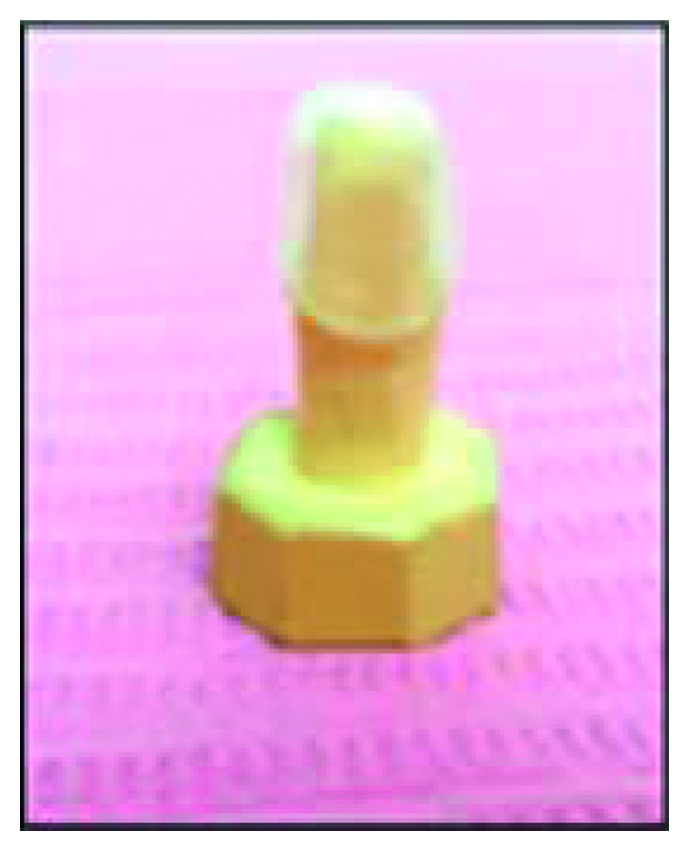
Coping former.

For standardization of the metal coping thickness and contour with that of CAD/CAM zirconia coping, duplicating addition silicon was used to make an impression for one of the zirconia copings; then, hard stone type IV was poured, and the stone die (Figure 5) was trimmed. And with the aid of the vacuum forming machine, a transparent template was fabricated on the stone die using 0.02 inch thickness under vacuum pressure and was trimmed after cooling with a scissor to a level approximately 2 mm cervical to the finish line to act as a vertical stop and vented to get a coping former that will be used later for resin pattern fabrication from self-curing acrylic resin (PATTERN RESIN™LS) (Figures 6 and 7).

Ten resin copings were sprued, invested using phosphate-bonded investment, and then placed inside the preheating furnace where burn out was done followed by casting of the nickel-chromium alloy according to the manufacturer's instructions. After divesting the metal copings, the sprues were cut and the copings were sandblasted and then finished and ultrasonically cleaned to get rid of all investment traces. Finally, the metal copings were seated onto their corresponding epoxy resin dies and checked for thickness and proper fit.

Ten nickel-chromium copings were veneered with Ceramco 3 using the layering technique, and as means of standardization, the split silicon molds that were used for fabrication of zirconia all-ceramic crowns were used again for fabrication of metal ceramic crowns with the same technique. After checking the metal ceramic crowns thickness and fit, the crowns were finally finished and glazed according to the manufacturer's instructions.

#### 2.2.6. Cementation of the Crowns

**Figure 8:**
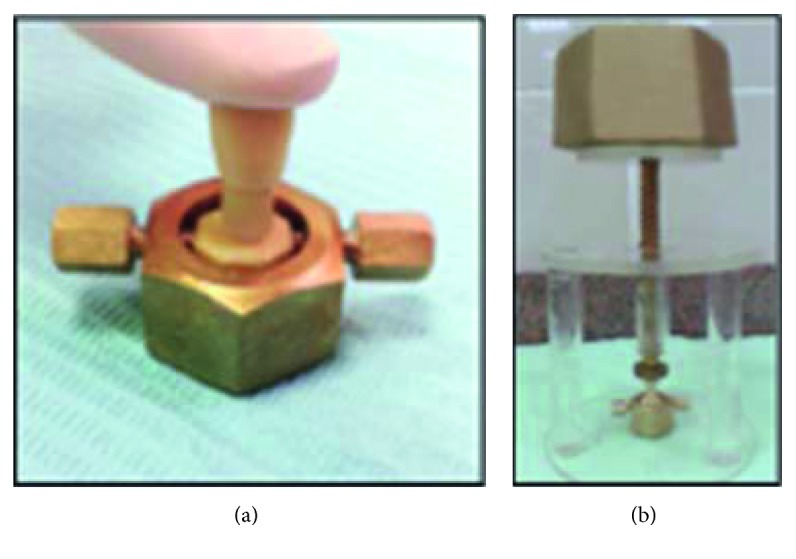
Seating the crown after cementation (a) under finger pressure and (b) under static load of 5 kg.

Self-curing luting resin with the light-curing option (Multilink N system pack) was used for luting all of the crowns according to manufacturer's instructions, and the restoration was seated in place and fixed by finger pressure for 15 seconds and then under static load of 5 kg for 10 minutes [15] (Figures 8(a) and 8(b)). Excess resin cement was removed immediately with a scaler, and light curing (quarter technique) was used; all margins were light cured again for 20 seconds per each surface under the static load device. Finishing and polishing of the cement at the crown margins were carried out using both silicon rubbers and discs.

#### 2.2.7. Testing

**Figure 9:**
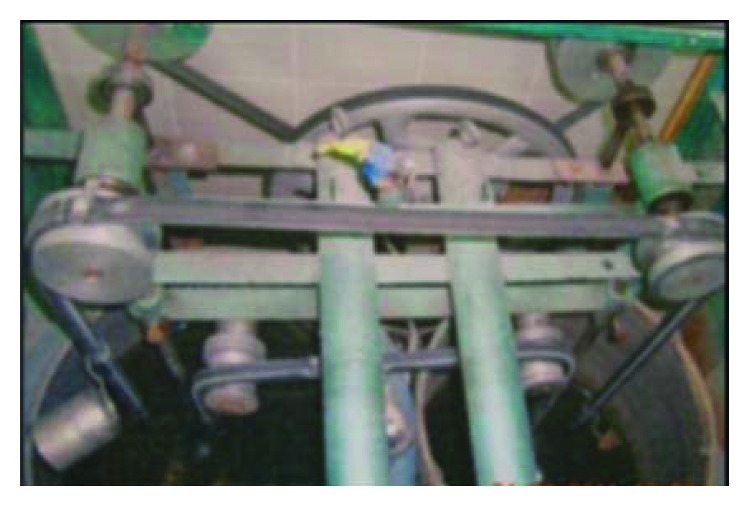
Specimens inside the thermal cycling machine.

*(1) Thermal Cycling*. Thermal cycling is the laboratory model used to mimic oral temperature changes. Each specimen was placed into a specially designed tray suspended from the arm of a thermal cycling machine. The specimens were subjected to 300 thermal cycles corresponding to six months of clinical service [16, 17]; thermal cycles were performed mechanically with a machine that transfers the specimens between two temperature-controlled water bath (the bath temperatures were adjusted at 5°C and 55°C with a dwell time of one minute in each bath and relaxation period of 30 seconds in air between the two baths) (Figure 9).

*(2) Cyclic Loading*. Each specimen was embedded in an acrylic block of size 19 × 19 × 20 mm, and then cyclic loading was performed using a specially designed custom-made load cycling machine where the loading stylus ended with a beveled metal rod with a dimension of 7 mm × 2 mm to apply load at 135° angle to the long axis of the tooth at the palatal surface of the crown, 2.5 mm from the incisal edge to simulate class I occlusion relationship with the antagonist tooth [18–22].

Cycling loads in this study were corresponding to 6 months of clinical service. Accordingly, samples were exposed to 120,000 mechanical cycles [23]. The load cycle was estimated by movement of a small metal projection attached to the upper metal arm within a groove in the oval-shaped acrylic block that allows the metal stylus to move up and down with a frequency of 1.7 Hz.

**Figure 11:**
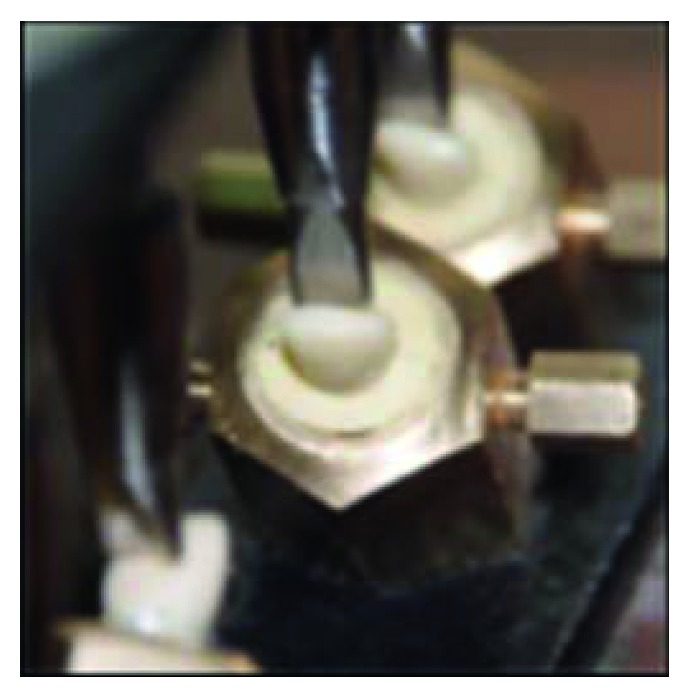
Loading stylus applying load onto the palatal surface of the crown 135° to the long axis of the tooth during the cyclic loading test.

**Figure 12:**
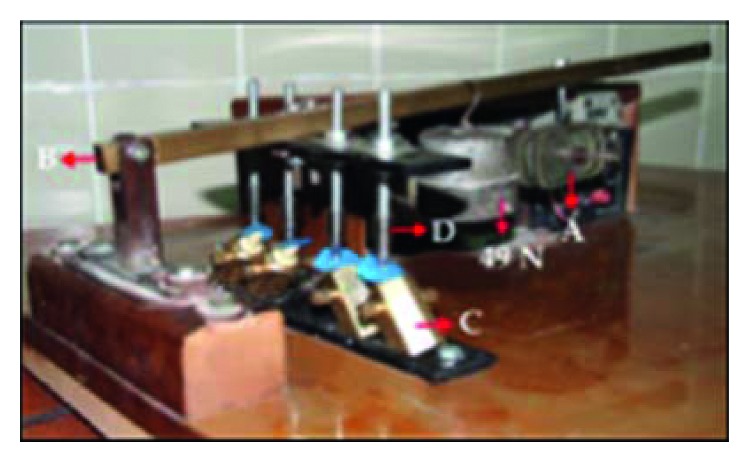
Specimens subjected to cyclic load. A, motor drive connected to an oval-shaped acrylic block with a metal rod; B, the metal arm fixed to the base of the machine from the one end and the other end carrying a metal block 49 N; C, four custom-made copper molds, carrying the specimens; D, four metal stylus, each one ended with 7 mm × 2 mm diameter beveled metal rod.

A rubber sheet of 0.5 mm thickness was inserted between the metal stylus and the crown to prevent sharp contacts, to distribute the applied force equally and to represent the consistency of the food substance [24] (Figures 11–12).

**Figure 13:**
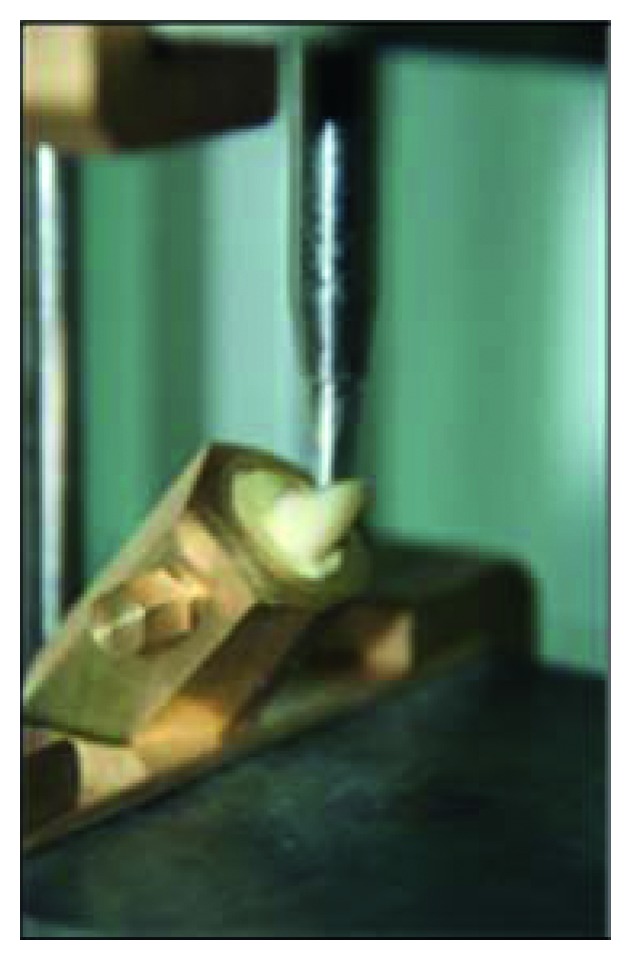
Loading stylus applying load onto the palatal surface of the crown 135° to the long axis of the tooth during the fracture resistance test.

*(3) Fracture Resistance Test*. To determine the fracture resistance of the specimens, a universal testing machine was used (Figure 13). Each embedded specimen was inserted into a copper mold of the lower jaw of the testing machine which was fixed at 45° degrees to the horizontal plane. A vertical compressive load was applied with a cross-head speed of 0.5 mm/min [25, 26] by means of a 7 mm × 2 mm beveled metal rod attached to the upper jaw of the testing machine, adjusted at 135° to the long axis of the tooth, and directed toward the palatal surface of the crown 2.5 mm from the incisal edge to simulate class I occlusion relationship with the antagonist tooth, and a rubber sheet was placed in between [18–22].

The load was applied on each specimen until catastrophic failure occurred. Catastrophic failure was defined as exhibition of visible cracks and events of chipping or fracture [27]. The failure load was recorded in newton on a reading monitor for each sample, and the fractured crowns were examined under the stereomicroscope to determine the fracture mode of each sample which was classified as following [18, 27, 28]:

  Mode I: visible cracks in the crown.
  Mode II: veneer chipping.
  Mode III: bulk fracture of the crown.

#### 2.2.8. Statistical Analysis

Data were collected and revised and coded and fed to statistical software IBM SPSS version 20. The given graphs were constructed using Microsoft excel software.

All statistical analysis was done using two-tailed tests and an alpha error of 0.05. *P* value less than or equal to 0.05 was considered to be statistically significant, and the following statistical tests were used:

- Descriptive statistics included the mean with standard deviation and percent to describe the scale and categorical data, respectively
- Analysis of numeric data included the independent sample *t*-test and two-way analysis of variance (ANOVA)
- Analysis of categorical data included the Mont Carlo exact test and Fisher's exact test

## 3. Results and Discussion

Most of the recommendations for a clinically relevant in vitro load-to-fracture test for ceramic restorations described by Kelly [29] and Rekow et al. [30] were followed in this study, including using a die material with elastic modulus similar to dentin, preparing the dies according to clinical guidelines, testing ceramic crowns with clinically relevant dimensions, applying fabrication techniques that closely anticipate laboratory and clinical procedures, and using a reliable commonly used luting cement [31].

After thermal and load cycling, no signs of fracture were detected in the specimens of each group. Signs of fracture were demonstrated by the specimens of each group only in the fracture resistance test, and the load at which fracture occurred for each specimen was recorded in newton.

### 3.1. Fracture Resistance

**Figure 14:**
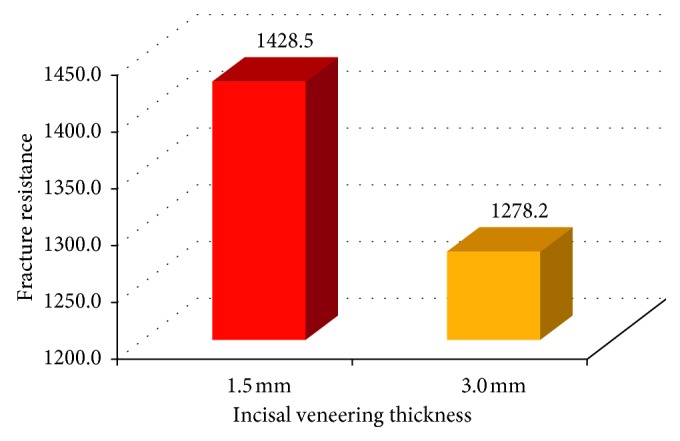
Bar chart showing fracture resistance of the two incisal veneering thicknesses in CAD/CAM zirconia all-ceramic groups.

The results revealed that there was a significant difference in the fracture resistance between the two incisal veneering thicknesses in both groups. For CAD/CAM zirconia all-ceramic group, there was a significant difference at (*P* < 0.05) between the fracture resistance of subgroups Ia and Ib where *t* = 4.2. CAD/CAM zirconia ceramic crown specimens with 1.5 mm incisal veneering thickness showed higher mean values (1428 ± 72.2 N) than those with 3 mm thickness (1278.2 ± 33.3 N) (Figure 14).

**Figure 15:**
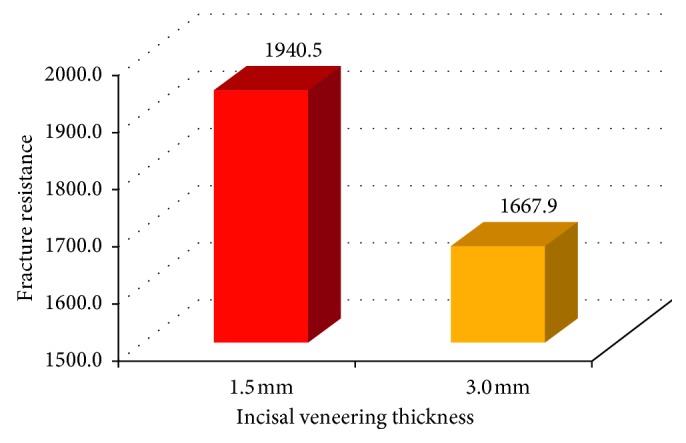
Bar chart showing fracture resistance of two incisal veneering thicknesses in the nickel-chromium metal ceramic group.

For the metal ceramic group, a significant difference was also reported at *P* < 0.05 between the fracture resistance of subgroups IIa and IIb where *t* = 3.5. The mean values for specimens with 1.5 mm veneering thickness was 1940.5 ± 153.7 N which is higher than those with 3 mm veneering thickness (1667.9 ± 80.2 N) (Figure 15).

This finding is in accordance with Swain [32] who reported in a similar study that thick layers of veneering ceramic on framework with low thermal diffusivity, such as Y-TZP, promoted the development of high tensile interior residual stresses resulting in chipping. Also, Mainjot et al. [33] stated in his study that veneering thickness influenced in an opposite way the favorable residual stress profile in both metal- and zirconia-based structures.

**Figure 16:**
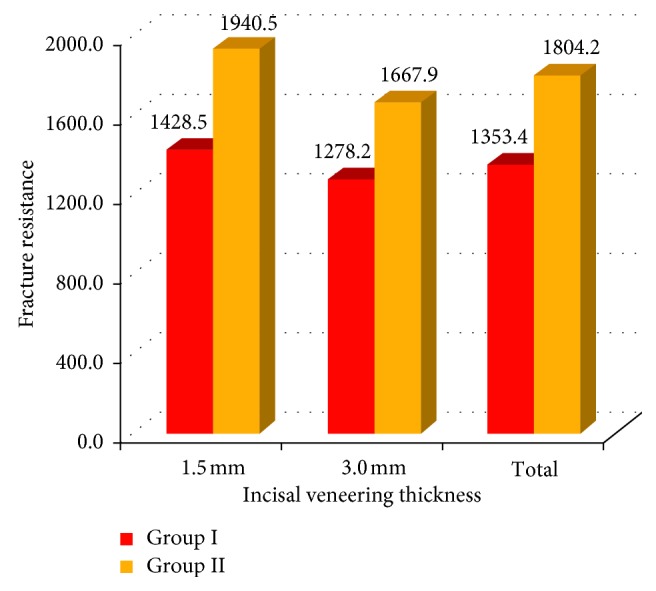
Bar chart showing fracture resistance of the two studied groups.

The results revealed that there was a significant difference (*P* < 0.05) in the fracture resistance between the two groups. The metal ceramic group showed a higher mean value (1804.2 ± 184.4) than the mean value of the CAD/CAM zirconia all-ceramic group (1353.4 ± 95.3) (Figure 16) (Table 3).

**Figure 17:**
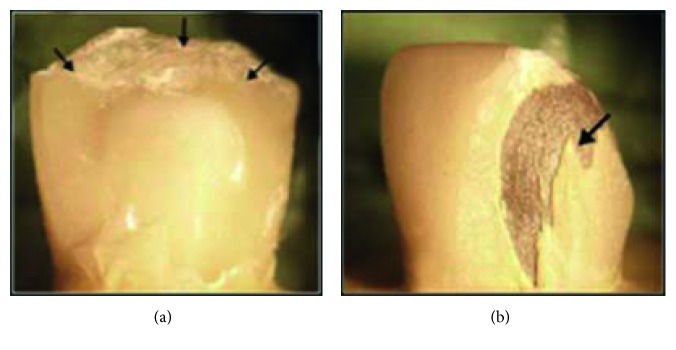
Mode II (veneer chipping) under stereomicroscope: (a) all-ceramic; (b) metal ceramic.

This finding is similar with other studies conducted by Silva et al. [34] and Hientze et al. [35] and can be explained by the different properties of the metal and the zirconia core, such as the modulus of elasticity, hardness, and toughness. Moreover, the composition of metal ceramic veneer has leucite reinforcing crystals to improve fracture resistance and to create thermal expansion compatibility with metal substructures. However, for zirconia coping, leucite is not needed in the zirconia veneering ceramic which is a multiphase mixture of glass compositions. Accordingly, the fracture surfaces of the porcelain for zirconia veneer were very flat and glassy in appearance (Figure 17).

Regarding the failure load values in this study, both groups demonstrated failure load values around 1500 N (Table 4) and this finding was in agreement with similar studies conducted by Sundh et al. [36], Guazzato et al. [37], and Yoon et al. [38]. However, it is in contradiction with Kelly [29] who reported failure load values around 600 N, where the load-to-failure test was performed under wet conditions in which a phenomenon termed “chemically assisted crack growth” or “static fatigue” was applied to simulate the active participation of water in intraoral conditions, while in the current study, the load-to-failure test was performed under dry conditions.

According to Waltimo and Könönen study, the mean maximum incisive force of the anterior teeth was 263 N for men and 243 N for women [39]. All of the fracture loads in the present study were much higher than those of the reported maximum incisive forces, as reported by other similar studies [40]. Also, the failure load values exceeded the maximum force recorded during clenching efforts (approximately 216 to 890 N) [41].

### 3.2. Modes of Fracture

**Figure 18:**
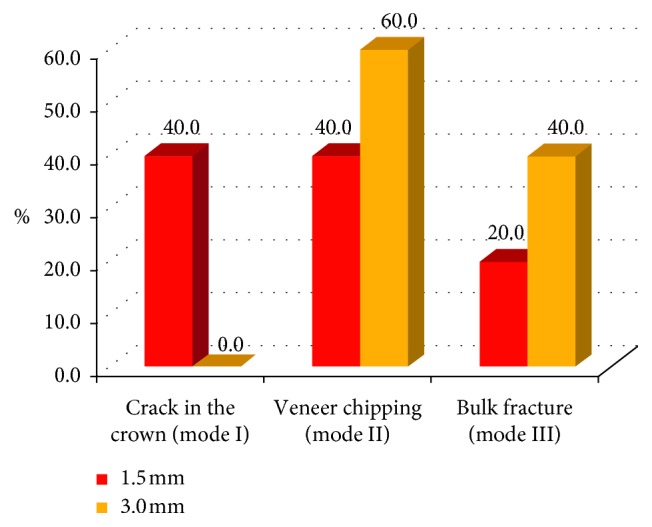
Bar chart showing the mode of fracture of each veneering thickness in both groups.

It was found that there was no significant difference in the modes of fracture demonstrated by the two groups in the two incisal veneering thicknesses since 50% of the total specimens demonstrated Mode II (veneer chipping). However, 35% demonstrated Mode I (visible crack), and only 15% demonstrated Mode III (bulk fracture) (Figure 18) (Table 3).

**Figure 19:**
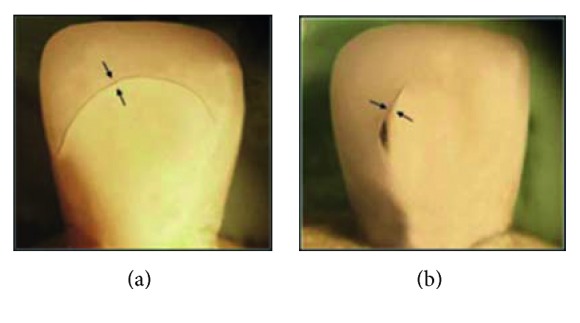
Mode I (visible crack) detected under stereomicroscope: (a) all-ceramic; (b) metal ceramic.

Veneer chipping demonstrated by 50% of total specimens (Figure 19); this finding is in accordance with Rosentritt et al. [42] and Silva et al. [34] who reported that the predominant failure pattern in both CAD/CAM zirconia and metal ceramic crowns was veneer chipping.

The chip size in the current study was much greater with zirconia crowns, creating more unacceptable defects; the surfaces where the veneering porcelain were delaminated from the core appeared smooth with no residual porcelain detected, and these observations were similar to previous studies [30, 43].

Visible crack demonstrated by 35% of total specimens (Figure 17); this finding is in accordance with previous studies [44, 45] which reported that cracks, initiating from the loading area, became evident long before the obvious event of fracture in all-ceramic restorations and cone cracking which are vertical planes where shear stresses are revealed. Porcelain cracks have also been ascribed to tensile stresses arising from internal or external flaws and in a fractographic analysis of failed clinical zirconia crowns, and heavy occlusal wear spots were observed at the failure origins [46].

**Figure 20:**
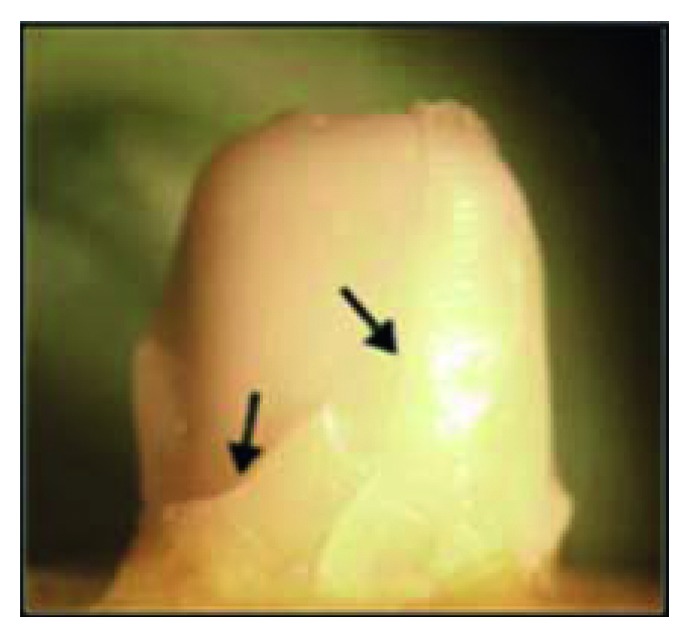
Mode III (bulk fracture) in all-ceramic specimen.

Bulk fracture demonstrated by 15% of total specimens and only by the CAD/CAM zirconia all-ceramic specimens (Figure 20), and this finding is similar with Çehreli et al.'s study [47] who reported rarely occurring core fracture of CAD/CAM zirconia crowns which indicate the high strength of the coping material.

Observations in this study and other studies revealed that fracture or chipping of veneering porcelains can be either a fracture of the porcelain itself or a fracture originating from the interfaces between the coping and porcelain [48]. A structural explanation for such behavior includes the mismatch of the coefficient of thermal expansion (CTE) between zirconia coping and veneering porcelain and residual stresses in veneering porcelain during the cooling process [49, 50]. The CTE of veneering porcelain must be slightly lower than that of zirconia coping to place veneering porcelain under compression and to increase bond strength between veneering porcelain and zirconia coping. Then, there will be less risk of crack development. A mismatch of the CTE between the zirconia coping and veneering porcelain produces stress fields throughout the entire restoration. Therefore, zirconia restorations should be slowly cooled to slightly below the glass transition temperature of the veneering porcelain to avoid the onset of residual tensile stresses [51].

Poor wettability of feldspathic porcelain is also another material specific factor for veneering chipping and is influenced by some processing parameters such as the roughness of the core surface and the atmosphere in which the feldspathic veneer was fused on the dental zirconia core. Such factors have a huge effect on the bond strength between zirconia and the overlying porcelain [52]. Coping design is also a crucial contributing factor that has been investigated in other studies in order to address possible reasons for the chipping issues [40, 53].

Regarding the fracture pattern, in the present study, the fracture pattern was extending from the lingual to the labial surface, and this finding is in consistent with Geminiani et al. [54] and Kim et al. [55] who reported a similar fracture pattern in anterior ceramic restorations. Such finding could be attributed to the fact that flaw population (size, number, and distribution) can be related to the type of material and the fabrication process. In the current study, a multilayer procedure was used to veneer the copings of both groups, and such procedure is sensitive and subjected to variability due to the individual building and firing which in turn could change the stress distribution pattern into a completely different and complex pattern that makes its performance to be hardly predictable in clinical situations (Table 5).

The results revealed that there was no interaction between the tested crown material and the veneering thickness (*P*=0.494), no interaction between the tested crown material and the modes of fracture (*P*=0.634), and also no interaction between the veneering thickness and the modes of fracture (*P*=0.737).

Increased numbers of specimens could have reduced the influence of data variations on the statistical outcome. Furthermore, as with any in vitro study, it remains unclear as to what extent the results may be different in a clinical setting. Higher numbers of loading cycles may be required to represent longer service time.

Scanning electron microscopic (SEM) investigation of the initiation and propagation direction of the cracks and failures would have been beneficial in studying the association between the defect at the loading point and the crack or fracture lines. Use of finite element analysis (FEA) also would be helpful in investigations of stress distribution to evaluate the mechanical behavior of restorations.

## 4. Conclusions

The current study [56] suggests that the fracture resistance of the nickel-chromium metal ceramic group was significantly higher than that of the CAD/CAM zirconia all-ceramic group; adding to this, the fracture resistance of crowns with 1.5 mm incisal veneering thickness was significantly higher than those with 3 mm incisal veneering thickness in both groups. High failure load values were demonstrated by the specimens in this study, which suggests sufficient strength of both incisal veneering thickness in both crown systems to withstand clinical applications; however, the fracture patterns still underline the requirement of a core design that supports a consistent thickness of the veneering ceramic, and it is recommended to conduct long-term prospective clinical studies to confirm findings reported in the present study.

## Figures and Tables

**Figure 10:**
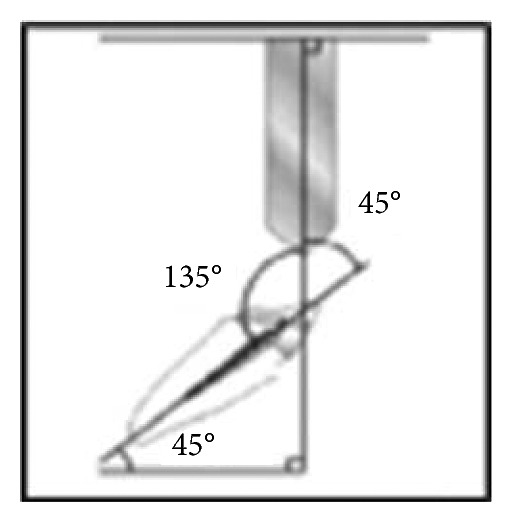
Schematic representation of load at an angle of 135° to the root long axis (45° to the horizontal plane).

**Table 1:** Crown materials used in this study.

System	System	Metal ceramic
(1) Coping material	Zirkonzahn^*∗*^ partially sintered zirconia blank	Nickel-chromium alloy art alloy BF^*∗∗*^
Fabrication technique	CAD/CAM	Lost wax technique
Composition	Main component is zirconium dioxide (ZrO_2_) + yttrium oxide (Y_2_O_3_ 5%) Hafnium oxide (Hf_2_O_3_ < 2%) Aluminium oxide + silicon oxide < 1%	Ni 62% Cr 23.5% Mo 10% Components less than 2% are Si, Mn, Nb, and Ti
(2) Veneering material	Porcelain for zirconia Ceramco®PFZ^*∗∗∗*^	Porcelain for nickel-chromium alloy Ceramco 3^*∗∗∗*^
Fabrication technique	Layering technique	Layering technique
Composition	Feldspathic porcelain containing no leucite	Feldspathic porcelain containing 0.30 volume fraction leucite

^*∗*^Zirkonzahn, GmbH, Bruneck, Italy. ^*∗∗*^Mesa di sala Giacomo and C-Snc-PI: 00623390176, Travagliato (BRESCIA), Italia (http://www.mesaitalia.com). ^*∗∗∗*^Dentsply International Inc., York, USA (http://www.ceramco.com and http://www.densply.com).

**Table 2:** Grouping of the specimens.

Groups	Group I		Group II	
Crown material	CAD/CAM zirconia all-ceramic (0.5 mm coping thickness)		Nickel-chromium metal ceramic (0.5 mm coping thickness)	
Number of specimens	10		10	
Subgroups	Ia	Ib	IIa	IIb
Number of specimens	5	5	5	5
Incisal veneering porcelain thickness	1.5 mm	3.0 mm	1.5 mm	3.0 mm

**Table 3:** Comparison between modes of fracture of total specimens in each incisal veneering thickness.

Mode of fracture	Incisal veneering thickness				MCP
	1.5 mm		3.0 mm		
	No.	%	No.	%	
Crack in the crown (Mode I)	5	50.0	2	20.0	0.364
Veneer chipping (Mode II)	4	40.0	6	60.0	
Bulk fracture (Mode III)	1	10.0	2	20.0	

MCP: *P* value based on Mont Carlo exact probability.

**Table 4:** Comparison between the two studied groups according to the fracture resistance in each incisal veneering thickness and in both thicknesses.

Incisal veneering thickness	Fracture resistance	Group		*t*	*P*
		Group I	Group II		
1.5 mm	Minimum	1339.0	1707.0	6.7	0.000^*∗*^
	Maximum	1535.0	2100.0		
	Mean	1428.5	1940.5		
	SD	72.2	153.7		

3.0 mm	Minimum	1230.0	1562.6	10.0	0.000^*∗*^
	Maximum	1316.0	1770.0		
	Mean	1278.2	1667.9		
	SD	33.3	80.2		

Total	Minimum	1230.0	1562.6	7.0	0.000^*∗*^
	Maximum	1535.0	2100.0		
	Mean	1353.4	1804.2		
	SD	95.3	184.4		

*t*, independent samples *t* test; ^*∗*^*P* < 0.05 (significant).

**Table 5:** Two-way analysis of variance (ANOVA) test of significance for comparing the mean fracture load by groups, incisal veneering thickness, and modes of fracture.

Source	*F*	*P*
Group	75.7	0.000^*∗*^
Thickness	14.6	0.003^*∗*^
Modes of fracture	1.2	0.344
Group *∗* thickness	0.5	0.494
Group *∗* fracture	0.2	0.634
Thickness *∗* fracture	0.3	0.737

## Data Availability

The experimental, observational, simulation, and compiled data used to support the findings of this study are included within the article.
