# Corrigendum to “Effect of Incisal Porcelain Veneering Thickness on the Fracture Resistance of CAD/CAM Zirconia All-Ceramic Anterior Crowns”

**DOI:** 10.1155/2020/2761094

**Published:** 2020-07-15

**Authors:** Noha Badran, Sanaa Abdel Kader, Fayza Alabbassy, Amir Azer

**Affiliations:** ^1^Conservative Dentistry Department, Fixed Prosthodontic Division, Faculty of Dentistry, Alexandria University, Alexandria City, Egypt; ^2^Conservative Dentistry Department, Faculty of Dentistry, Alexandria University, Alexandria City, Egypt; ^3^Dental Biomaterials Department, Faculty of Dentistry, Alexandria University, Alexandria City, Egypt

In the article titled “Effect of Incisal Porcelain Veneering Thickness on the Fracture Resistance of CAD/CAM Zirconia All-Ceramic Anterior Crowns” [1], Dr. Amir Azer was missing from the authors' list. Dr. Azer edited and commented on the manuscript and approved the final version. He was a main supervisor on the Master's thesis from which this work derived and he contributed to the study planning through his static loading device, which was used in the cementation procedure of the specimens. The corrected authors' list is shown above.
